# Coping mediates the relationship between sense of coherence and mental quality of life in patients with chronic illness: a cross-sectional study

**DOI:** 10.1007/s11136-018-1845-0

**Published:** 2018-04-05

**Authors:** Marja-Leena Kristofferzon, Maria Engström, Annika Nilsson

**Affiliations:** 1 Department of Health and Caring Sciences, Faculty of Health and Occupational Studies, University of Gävle, Kungsbäcksvägen 47, 801 76 Gävle, Sweden; 2 Department of Public Health and Caring Sciences, Uppsala University, Uppsala, Sweden; 3 Nursing Department, Medicine and Health College, Lishui University, Lishui, China

**Keywords:** Coping, Chronic illness, Quality of life, Sense of coherence, Coping effectiveness

## Abstract

**Purpose:**

The aim of the present study was to investigate relationships between sense of coherence, emotion-focused coping, problem-focused coping, coping efficiency, and mental quality of life (QoL) in patients with chronic illness. A model based on Lazarus’ and Folkman’s stress and coping theory tested the specific hypothesis: Sense of coherence has a direct and indirect effect on mental QoL mediated by emotion-focused coping, problem-focused coping, and coping efficiency in serial adjusted for age, gender, educational level, comorbidity, and economic status.

**Methods:**

The study used a cross-sectional and correlational design. Patients (*n* = 292) with chronic diseases (chronic heart failure, end-stage renal disease, multiple sclerosis, stroke, and Parkinson) completed three questionnaires and provided background data. Data were collected in 2012, and a serial multiple mediator model was tested using PROCESS macro for SPSS.

**Results:**

The test of the conceptual model confirmed the hypothesis. There was a significant direct and indirect effect of sense of coherence on mental QoL through the three mediators. The model explained 39% of the variance in mental QoL.

**Conclusions:**

Self-perceived effective coping strategies are the most important mediating factors between sense of coherence and QoL in patients with chronic illness, which supports Lazarus’ and Folkman’s stress and coping theory.

## Introduction

Chronic illnesses, such as heart and kidney failure and neurological diseases, e.g., multiple sclerosis, stroke, and Parkinson’s disease, affect several dimensions of everyday life. Patients need to handle symptoms, treatment, functional impairments [1–7], comorbidity [2, 8, 9], and unpredictability with regard to symptoms and disease progression [2, 10]. How persons with chronic illness cope with limitations and what coping resources they have and use can impact their quality of life (QoL) [1, 3, 4, 10–14]. Social isolation [2, 5], psychological distress [2, 4–6, 15], and loss of sense of control [2] are some of the mental consequences described in the literature.

Patients with chronic illness have narrated that physical limitations, symptoms, chronicity, and the progressive nature of the illness as well as the impact of conditions on daily activities and relationships have a negative effect on their QoL [16–18]. They attempted to handle shortcomings to preserve an independence and normality in their life, activated personal resources, and looked for new sources of well-being [17]. The present study has examined relationships between coping resources, coping, and mental QoL by testing a conceptual model based on Lazarus’ and Folkman’s stress and coping theory [19].

## Theoretical framework

Living with chronic illness may threaten health and QoL and is influenced by the person’s coping strategies and personal resources [14, 19]. A personal resource introduced by Antonovsky is the concept of sense of coherence, which refers to the extent to which persons believe that life events they encounter are comprehensible, manageable, and meaningful. Comprehensibility means that events are understandable and predictable. Manageability refers to the extent persons consider their personal resources adequate in meeting the demands. Meaningfulness is the motivational dimension of sense of coherence and refers to the extent to which persons believe the demands are worthy of engagement. According to Antonovsky, persons need to identify and use general resistance resources—e.g., material resources, knowledge, confidence, and consistent belief and value systems—to develop a strong sense of coherence. Persons with a stronger sense of coherence are better able to cope with stressful situations and more likely to perceive good QoL [20].

Lazarus and Folkman describe coping as a process in which persons use cognitive and behavioral efforts to manage stressful situations. Coping is a psychological process focused on persons’ own interpretations of their situation. In focus here is persons’ self-evaluation of what coping strategies they find effective [19]. Lazarus means that coping strategies should be divided into two groups—emotion-focused or problem-focused—with respect to the function of the respective coping strategies [21]. Emotion-focused coping is used when persons try to regulate emotions the situation causes, e.g., using relaxation, meditation, or avoiding information. These strategies are used when persons need to avoid or escape from the situation. Problem-focused coping refers to strategies persons use to solve or alter the stressful situation or themselves, e.g., planning what to do or seeking information. These strategies are used when persons feel they have the ability to manage the situation [22, 23]. The two coping functions (emotion- and problem-focused) should not be treated as two independent types of coping, because in most stressful situations, such as living with chronic illness, they complement each other [14, 21].

## Relationships between sense of coherence, coping, and QoL in chronic illness

Lower sense of coherence has been shown to be related to higher use of emotion-focused coping in patients with chronic heart failure [1, 12] or end-stage renal disease [12], whereas higher sense of coherence was related to higher QoL [12]. Sense of coherence has been found to predict coping and QoL in patients with Parkinson’s disease [24]. More use of problem-focused coping was associated with better QoL in patients with chronic heart failure [1, 11], Parkinson’s disease [25], and stroke [26], whereas more use of emotion-focused coping was associated with poorer QoL [11, 12, 25, 27]. Positive correlations between coping and QoL have been found in patients with stroke [5], multiple sclerosis [13], and end-stage renal disease [7, 28]. Person factors such as age [1, 13, 14, 29, 30], gender [1, 10, 13, 14, 30–33], educational level [14, 15], economic status [14], and comorbidity have been shown to be related to coping [34].

A systematic review (of studies mainly on samples of patients with chronic illnesses) showed positive influences of sense of coherence on QoL [35]. Coping has been proposed to mediate between personal resources, such as sense of coherence, and QoL [14, 19]. Most of the studies found have investigated associations between sense of coherence, coping, and QoL in patients with chronic illness using regression analyses [1, 7, 12, 13, 24, 25, 28]. None of them has tested coping as a mediator between sense of coherence and QoL.

## Conceptual model

**Fig. 1:**
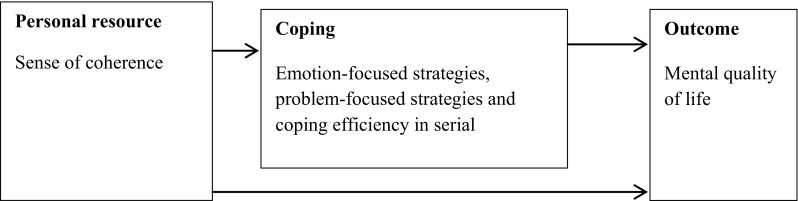
An overview of the conceptual model in the present study based on Lazarus’ and Folkman’s stress and coping theory [19]. The arrows in the figure show the flow of the hypothesized influence of the variables tested in the present study. Sense of coherence is hypothesized to influence both directly and indirectly (through coping) on the outcome. Person factors (age, gender, educational level, comorbidity, economic status) are treated as covariates in the conceptual model

The conceptual model used in the present study and displayed in Fig. 1 is based on Lazarus’ and Folkman’s stress and coping theory [19]; it tested the influences of sense of coherence and coping on mental QoL. Coping is a process that concerns how persons think, feel, and act in specific stressful situations, and it aims to reduce perceived stress and negative emotions. Person factors (e.g., age, gender, educational level, comorbidity, and economic status) and personal resources (e.g., sense of coherence) are aspects related to coping and can be seen as prerequisites in the coping process. QoL is viewed as an outcome of the coping process. The level of QoL depends on how efficient the person with chronic illness perceives the used coping strategies to be. In the conceptual model, coping is seen as a mediator between sense of coherence and mental QoL [14, 19].

## Aim

The aim of the present study was to investigate the relationships between sense of coherence, emotion-focused coping, problem-focused coping, coping efficiency (perceived efficiency in managing physical, psychological, social, and existential aspects of daily life), and mental QoL in patients with chronic illness.

A model was tested based on Lazarus’ and Folkman’s stress and coping theory. The model tests the specific hypothesis that sense of coherence has a direct and indirect effect on mental QoL mediated by emotion-focused coping, problem-focused coping, and coping efficiency in serial adjusted for age, sex, educational level, comorbidity, and economic status.

## Method

### Design

The present cross-sectional study had a correlational design and included self-reported data from patients with chronic illnesses.

### Setting and sample

The sample included patients from one regional and one rural hospital, situated in a region in central Sweden with about 276,500 inhabitants. Patients with chronic heart failure, end-stage renal disease, and neurological diseases such as multiple sclerosis, stroke, and Parkinson’s disease were taken from the International Statistical Classification of Diseases and Related Health Problems, 10th Revision, in 2011 by a head administrator from the regional hospital. Moreover, two of the authors (M-LK, AN) selected the patients, who matched the age range, from the list they got from the head administrator. Those meeting the following inclusion criteria were asked to participate: 18–85 years of age, have had the disease for more than 3 months and understood the Swedish language. Out of 1029 patients, 348 participated (34%). Thirty-five patients declined to participate and the rest were non-responders. Because 60 patients had incomplete questionnaire replies, 292 were included in the analysis.

### Data collection

Data were collected from October 2011 to March 2012 using three self-administrated questionnaires. In addition, data were collected on person characteristics such as age, gender, civil status, educational level, economic situation, work conditions, living area, and comorbidity.

Sense of coherence was measured using the 13-item short form of the Sense of Coherence scale [20, 36]. It consists of 13 items, and responses are made on a 7-point scale. After reversing five of the items, a total score is calculated that ranges from 13 (low sense of coherence) to 91 (high sense of coherence). The total scale was used in the present study, and the internal consistency was 0.82. In previous studies, the scale has been considered valid and reliable [37, 38] and Cronbach’s alpha values have been reported from 0.70 to 0.92 using SOC-13 and are cross culturally applicable [38].

Emotion-focused and problem-focused coping were measured using the Jalowiec Coping Scale (JCS-60) [34]. The total scale consists of 60 items, each of which describes a coping strategy a person can use in response to stress, which in the present study was having a chronic illness. The coping strategies are grouped into eight scales: (1) evasive, (2) fatalistic, (3) emotive, (4) palliative, (5) confrontative, (6) optimistic, (7) supportive, and (8) self-reliant. The first four scales measure emotion-focused coping, the remaining scales problem-focused coping. A 4-point scale was used to assess how often each coping category was used (0 = never, 1 = rarely, 2 = sometimes, 3 = often) [39–41]. Mean values were calculated for both emotion-focused and problem-focused coping; the internal consistency values for emotion-focused and problem-focused coping were 0.83 and 0.87, respectively. The JCS-60 has been tested for reliability and validity in previous research [12, 40].

Mental QoL was measured with the Mental Component Score (MCS) including four scales: vitality, social functioning, role-emotional, and mental health, from the Short-Form 36 Health Survey (SF-36), that is a generic health-related QoL measure. The response alternatives used were yes/no, 3-point (1 = yes, greatly limited to 3 = no, not at all limited), 5-point (e.g., 1 = not at all to 5 = very much), and 6-point scales (1 = all the time to 6 = none of the time). Scores for each scale were coded, summed, and transformed into a scale ranging from 0 (worst possible health) to 100 (best possible health) [42, 43]. In the present study, the internal consistency for the MCS was 0.72. The SF-36 has been shown to have good reliability (Cronbach’s alpha values 0.70–0.90) and validity for the eight scales and the two components (Physical Component Score and MCS). Construct validity has been supported by factor analysis and clinical group contrast analysis [42, 44, 45].

The variable “coping efficiency” is based on four items: perceived efficiency in managing (a) physical, (b) psychological, (c) social, and (d) existential aspects of daily life. Respondents indicate how well or how poorly they have coped with difficulties related to their chronic illness on a 5-point scale: very bad, bad, neither bad nor good, good, and very good. The four items have been used in previous research but not as a composite scale [32, 33, 46]. The internal consistency in the present study for “coping efficiency” was 0.81.

### Data analysis

Descriptive statistics were used to describe the study variables, and Cronbach’s alpha coefficients were calculated for the questionnaires used. Pearson product-moment correlations were used to test bivariate correlations between the main variables, and standard multiple linear regression analysis was used to test for regression model assumptions and the model’s explained variance.

To test our hypothesis concerning the direct and indirect effects of sense of coherence on mental QoL, we used serial multiple mediator models with procedures described by Hayes [47] and the PROCESS macro for SPSS. The indirect effect was tested in serial: emotion-focused coping, problem-focused coping, and coping efficiency, based on Lazarus’ and Folkman’s theory [19]. Included as covariates in the analysis were age, gender, educational level, comorbidity, and economic status. Point estimates unstandardized regression coefficients as well as bias-corrected and accelerated 95% confidence intervals (Boot CIs) are presented with the number of bootstrap samples set at 5000. Residuals were screened, histogram, normal P–P plot of regression residual, normal Q–Q plot of residual, and box-plots, and judged to be normally distributed. Data were checked for multicollinearity between independent variables using variance inflation factor (VIF) and all VIF values were below 10 (ranging from 1.1 to 3.3) indicating no problem [48]. Statistical analyses were performed using IBM SPSS Statistics 20. For all tests, the statistical significance level was set at *p* < 0.05.

### Ethical considerations

The study conformed to the ethical principles defined in the World Medical Association Declaration of Helsinki [49], and was approved by the regional ethical review board (reg. no. 2010/346). The questionnaires, a cover letter with information about the study, and a stamped reply envelope were sent by mail to the patients. Two reminders were sent out, and all data were coded using a subject number. When the patients completed and returned the questionnaires, this was considered to constitute their informed consent. All study patients were guaranteed confidentiality and were informed that their participation was voluntary (The Swedish Research Council 2011).

## Results

### Patient characteristics

The characteristics of the patients are presented in Table 1. There were 348 patients: 127 women and 221 men 22–85 years of age (mean age = 69, SD = 12.5). Most of the patients were married/cohabitant and retired. The answer alternative “Other” for the variable “Work conditions” includes that the person was a housewife/husband or unemployed. Most of the patients also had chronic heart failure as a primary disease.

**Table 1:** Characteristics of the patients

Variable	*n* (%)
Age	347
Mean (SD)	69.1 (12.54)
Range	22–85
Gender	348
Female	127 (36.5)
Male	221 (63.5)
Civil status	343
Married/cohabitant	230 (67.1)
Single	113 (32.9)
Educational level	339
Compulsory school	161 (47.5)
Senior high school	107 (30.7)
University	71 (20.9)
Economic situation	342
Very good	27 (7.9)
Good	121 (35.4)
Acceptable	162 (47.4)
Unsatisfactory	24 (7.0)
Very unsatisfactory	8 (2.3)
Work condition	345
Working	68 (19.7)
Retired	262 (75.9)
Other	12 (4.4)
Living area	340
Urban	125 (36.8)
Middle-sized town	93 (27.4)
Small town	50 (14.7)
Rural	72 (21.2)
Primary disease	348
Multiple sclerosis	52 (14.9)
Stroke	63 (18.1)
Parkinson	55 (15.8)
Chronic heart failure	124 (35.6)
End-stage renal	54 (15.5)
Comorbidities	345
Primary disease	120 (34.8)
More diseases than the primary disease	225 (65.2)

### Bivariate correlations between main study variables

Sense of coherence and coping efficiency were positively correlated with mental QoL, sense of coherence with coping efficiency, and emotion-focused coping with problem-focused coping. Emotion-focused and problem-focused coping were negatively correlated with mental QoL, sense of coherence, and coping efficiency (Table 2).

**Table 2:** Correlations between the main study variables and descriptive statistics (*n* = 303–341)

Variables	1	2	3	4	Mean (SD)	Scale
1. Mental quality of life	–				51.42 (11.20)	0–100
2. Sense of coherence	0.46***	–			65.91 (12.43)	13–91
3. Emotion-focused coping	− 0.39***	− 0.45***	–		0.95 (0.45)	0–3
4. Problem-focused coping	− 0.24***	− 0.23***	0.78***	–	1.44 (0.57)	0–3
5. Coping efficiency	0.59***	0.56***	− 0.40***	− 0.16**	3.48 (0.70)	1–5

Higher values indicate higher levels of the measured variables

*SD* standard deviation

**p < 0.01, ***p < 0.001

### Serial multiple mediator model

**Fig. 2:**
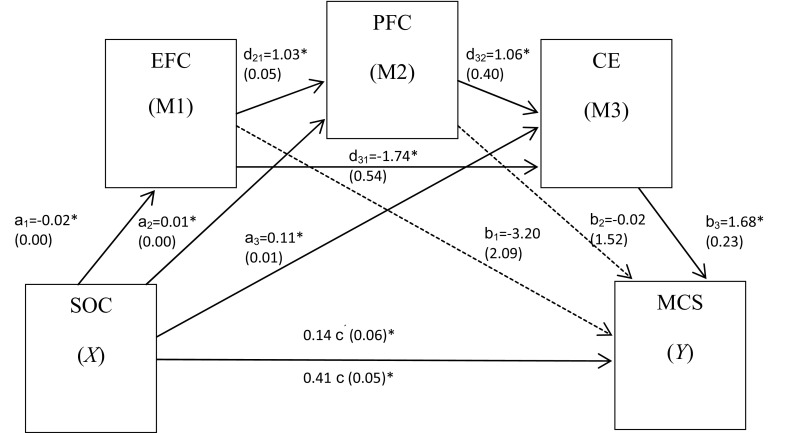
A schematic illustration of the effect of sense of coherence (SOC) through the mediators: Emotion-focused coping (EFC), problem-focused coping (PFC), and coping efficiency (CE), in serial, on mental quality of life [Mental Component Score (MCS)]. The coefficient *c* is the total effect between *X* and *Y* and *c*′ is the direct effect of *X* on *Y* while controlling for the three *M*. Values in the model are unstandardized regression coefficients and (standard errors). The variables are adjusted for age, gender, educational level, comorbidity, and economic status, *n* = 291. *p < 0.05 The serial multiple mediator model was tested using PROCESS procedure for SPSS [47]. Dotted lines represent non-significant paths

The results of the test of the conceptual model confirmed our hypothesis. The results showed a significant direct and indirect effect of sense of coherence on mental QoL through the three mediators (emotion-focused coping, problem-focused coping, coping efficiency) in serial [*a*_1_*d*_21_*d*_32_*b*_3_; unstandardized regression coefficient **−** 0.0312; Boot CIs − 0.060, − 0.009] controlling for age, gender, educational level, comorbidity, and economic status. Furthermore, there were significant indirect effects of sense of coherence on mental QoL through emotion-focused coping and coping efficiency in serial [*a*_1_*d*_31_*b*_3_; 0.0495; Boot CIs 0.020, 0.088] as well as through problem-focused and coping efficiency in serial [*a*_2_*d*_32_*b*_3_; 0.0130; Boot CIs 0.003, 0.030]. There were also significant indirect effects of sense of coherence on mental QoL through coping efficiency only [*a*_3_*b*_3_; 0.1783, Boot CIs 0.126, 0.247], but not for emotion-focused coping only [*a*_1_*b*_1_; 0.0542, Boots CIs − 0.010; 0.127] or problem-focused coping only [*a*_2_*b*_2_; − 0.0002; Boots CIs − 0.025, 0.025] or these two in serial [*a*_1_*d*_21_*b*_2_; 0.0004; Boots CIs − 0.057, 0.056]. The total indirect effect of sense of coherence on mental QoL was significant (all paths, see Fig. 2; 0.2639; Boot CIs 0.196, 0.352), i.e., an increase in 1 U in sense of coherence means an increase in mental QoL with 0.264 U.

The regression coefficient for sense of coherence on mental QoL (*c*)—controlling for age, gender, educational level, comorbidity, and economic status—was 0.406 (*p* < 0.001) decreasing to 0.142 (*c*′) when the three mediators were added (*p* = 0.012). The model itself could explain 39% of the variance in mental QoL (adjusted *R*^2^ = 0.368).

## Discussion

Our results support the proposed conceptual model, based on Lazarus’ and Folkman’s theory [19], suggesting that the coping strategies used and how effective they are perceived to be mediate the relationship between sense of coherence and mental QoL among persons with chronic illness. Evidence for the conceptual model adds knowledge to previous research, which has found relationships between sense of coherence, coping, and QoL in patients with chronic illness, but not tested coping as a mediator [1, 7, 12, 13, 24, 25, 28].

Emotion-focused and problem-focused coping separately or together did not mediate between sense of coherence and mental QoL. However, coping efficiency, together with coping strategies or alone, mediated between sense of coherence and mental QoL. The results indicate that persons with chronic illness who have a strong sense of coherence use few, but perceived effective, coping strategies to achieve good mental QoL. According to Antonovsky, those who are able to identify and use their own general resistant resources have a stronger sense of coherence and perceive better QoL. When persons with chronic illness perceive stress with regard to their illness and mobilize coping strategies they perceive to be effective, this can be seen as a general resistant resource (coping ability) [20, 35] that mediates between sense of coherence and QoL [14, 19].

Lazarus and Folkman have suggested that coping is situation-based and that persons use different coping strategies in different situations [19]. This need not be considered to diverge from the habitual coping strategies described by Coyne and Gottlieb [50], strategies patients with a chronic illness may develop over time. These are basic coping strategies used in particular types of stressful situations, thus forming a repertoire of coping options. This may be considered congruent with the present results, showing that use of few, but perceived effective, coping strategies in different stressful situations related to chronic illness resulted in better mental QoL.

The stress and coping theory proposed by Lazarus and Folkman [19] is a psychological theory based on adaptation to situations through regulation of emotions, which can be seen as a driving force behind coping with stress. Emotion-focused coping strategies focus on use of cognition to regulate emotions, whereas problem-focused strategies focus on actions [51]. Coping strategies are not inherently good or bad. What strategies are chosen depends on how controllable the person perceives the situation to be. Emotion-focused strategies are used in more uncontrollable situations, such as onset of disease or when new symptoms appear. Problem-focused strategies are used in more controllable situations, when persons know more about the impact of the disease. Often persons use both emotion-focused and problem-focused strategies in a stressful situation [14, 19]. Given Lazarus’ implicit claim that coping is based on emotions and often focuses on regulating emotions as in emotion-focused coping [51], we chose to put emotion-focused coping before problem-focused coping in our conceptual model. We also tested putting problem-focused coping before emotion-focused coping, and this did not change the significant results obtained.

A model developed by Paterson [52]—which seems to be consistent with the conceptual model suggested here, where the patient’s own interpretations in daily life are of vital importance—is the shifting perspectives model of chronic illness. It was developed in a meta-study of 292 primary studies using a qualitative approach to chronic physical illness. In their model, living with chronic illness is seen as an on-going, continually shifting process, containing both illness and wellness. Illness and wellness shift positions in the foreground or background depending on the situation at the moment. The perception of reality, not reality itself, provides the basis for how persons with chronic illness interpret and cope with their illness. When patients are diagnosed with a chronic disease or experience new symptoms, exacerbations or loss of function associated with existing disease, their primary perspective can shift from wellness to illness. In these situations, patients may need to use more emotion-focused strategies, e.g., avoid thinking about it, escape for a while by reading a book, watching a movie, crying, or feeling sorry for themselves, to put up with what has happened. These strategies can give them the energy to accept what has happened and may thus be perceived as effective. With this energy, they may begin to learn more about the disease and treatments, plan for the immediate future, create a supportive environment, and develop new skills. All these strategies are problem-focused. Gradually, their foregrounded perspective may shift from illness to wellness. When this occurs, patients are no longer centered around the chronic illness, but rather life itself.

The present results indicate that sense of coherence may play an important role as an internal resource mediated by coping in managing and controlling daily life among persons with chronic illness. Antonovsky has described sense of coherence as a relatively stable person characteristic. A person with a strong sense of coherence is more likely to judge a situation as comprehensible, manageable, and meaningful and to select appropriate coping strategies [20]. With regard to clinical practice, the question is how professionals can help to strengthen patients’ sense of coherence. There are promising results from an intervention performed recently in two groups with chronic illness, one in specialist care and one in community care. The intervention included discussions of health despite illness, the patient’s bodily knowledge, coping skills, health, and well-being, with a focus on strengthening sense of coherence among patients living with chronic illness. Included in the sample were, e.g., patients with Parkinson’s disease, multiple sclerosis, and chronic heart failure. The groups attended seven sessions over a 4-month period. Sense of coherence increased for the total group over time, and the change was greater among participants in community care. The intervention helped to strengthen patients’ coping abilities, and they were actively involved in identifying their own resources [53]. This is in line with what the National Board of Health and Welfare in Sweden has emphasized, namely, that health care practitioners need to increase their focus on persons’ resources and active involvement in promoting their own health and QoL [54].

The tested conceptual model supports Lazarus’ and Folkman’s theory [19], which points out the importance of discovering how patients themselves interpret their coping process. Taking into account the present results, it would seem to be important for health care practitioners to identify patients’ internal and external resources, such as coping abilities, beliefs, inner strengths, and social support. Moreover, practitioners should be aware of what coping strategies the individual patient uses in a specific situation and how effective the patient perceives the strategies to be. This knowledge is a resource for health care practitioners in care planning and interventions. It enables them to strengthen the patient’s existing general resistant resources and add resources that are lacking. Moreover, health care practitioners can empower the patient to identify and use his/her resources when interacting in person-centered care.

Social support has proven to be an important external resource when coping with chronic illness [14]. In further research, it would be interesting to test the interaction between sense of coherence and social support in the conceptual model as well as in prospective designs. Moreover, to test an intervention aimed at strengthening sense of coherence in patients with chronic illness seems to be relevant. In current research on positive psychology the role of gratitude has shown to affect SOC through positive reframing of life experiences [55]. The promising finding that gratitude has shown to enhance SOC, can be tested by future research and then be used in interventions.

Strengths of the study are that the conceptual model is based on a theory, validated instruments were used, and acceptable Cronbach’s alpha values obtained for our sample. Limitations are the methods used to select the study group and the low response rate, which make it difficult to generalize the results, as well as the cross-sectional design, which does not allow us to draw conclusions concerning causality. Social desirability bias for self-reported data can threat the validity, but recently reported findings showed that it might play a little role in well-being self-report measures [56].

## Conclusions

Self-perceived effective coping strategies are the most important mediating factors between sense of coherence and QoL in patients with chronic illness. This finding supports Lazarus’ and Folkman’s stress and coping theory. In patients with chronic illness, having few, but self-perceived effective, coping strategies may have a more positive affect on QoL than type of strategies used. The results of the present study need to be further tested in other groups of chronic illnesses and in longitudinal designs to generate knowledge on which to base more solid implications for clinical practice.
